# Ring quantum cascade lasers with twisted wavefronts

**DOI:** 10.1038/s41598-018-26267-x

**Published:** 2018-05-22

**Authors:** Rolf Szedlak, Thomas Hisch, Benedikt Schwarz, Martin Holzbauer, Donald MacFarland, Tobias Zederbauer, Hermann Detz, Aaron Maxwell Andrews, Werner Schrenk, Stefan Rotter, Gottfried Strasser

**Affiliations:** 1 Institute of Solid State Electronics & Center for Micro- and Nanostructures, TU Wien, Floragasse 7, 1040 Vienna, Austria; 2 Institute for Theoretical Physics, TU Wien, Wiedner-Hauptstraße 8-10/136, 1040 Vienna, Austria; 3 Austrian Academy of Sciences, Dr. Ignaz Seipel-Platz 2, 1010 Vienna, Austria

## Abstract

We demonstrate the on-chip generation of twisted light beams from ring quantum cascade lasers. A monolithic gradient index metamaterial is fabricated directly into the substrate side of the semiconductor chip and induces a twist of the light’s wavefront. This significantly influences the obtained beam pattern, which changes from a central intensity minimum to a maximum depending on the discontinuity count of the metamaterial. Our design principle provides an interesting alternative to recent implementations of microlasers operating at an exceptional point.

## Introduction

The field of non-Hermitian photonics has recently made considerable progress^1^. A topic that has attracted particular attention, was that of lasing at an exceptional point. Following a first theoretical proposal^2^ for how to induce such a non-Hermitian degeneracy in a laser just by tuning the pump profile, experiments quickly implemented this protocol successfully^3,4^. Interesting and counter-intuitive effects, such as a loss-induced suppression and revival of lasing, could thereby be linked to the presence of an exceptional point. Subsequent works demonstrated that parity-time symmetric lasers operating near an exceptional point are well-suited for single-mode operation^5,6^. Directly at an exceptional point, laser modes in a microdisk also have a well-defined sense of rotation^7^ - a property that has meanwhile also been observed in the experiment^8,9^. With appropriate outcoupling, such chiral modes spinning around in a microlaser can be made to emit an optical vortex beam carrying a well-defined optical angular momentum (OAM)^10^. Here we will investigate an alternative design principle for the creation of microlasers emitting beams with twisted wavefronts. Rather than carefully engineering the gain-loss profile in a ring laser as in ref.^5^ and ref.^10^, we work here with ring quantum cascade lasers (QCLs)^11^ whose substrate is patterned as a phase plate that twists the emitted laser beam.

The first QCL^12^ was based on a simple Fabry-Pérot resonator geometry consisting of a ridge waveguide with two cleaved facets. Over the years it has been demonstrated that modifications of the laser geometry can lead to a significant improvement of the emission beam. Properly tilting the emitting facet of the QCL ridge suppresses higher order modes, increases the output power and provides enhanced beam profiles^13,14^. A collimation of the far field profile has been shown using plasmonic antennas^15,16^, which are directly fabricated on the cleaved edge of the chip near the emitting facet. In contrast to these in-plane emitters, vertically emitting QCLs offer several advantages like on-chip testing and two-dimensional array integration. Due to their rather large emitting area they are capable of producing low-divergence laser beams. Since the first demonstration of a surface-emitting QCL^17^ based on a second order DFB grating, many other techniques have been successfully implemented. While photonic crystals^18–20^ exploit artificial periodic structures and provide single-mode emission, random lasers^21,22^ support coherent broadband radiation with a near diffraction-limited beam. Surface-emitting DFB QCLs operating in the symmetric mode exhibit a single-lobe far field pattern^23–25^. Furthermore, circular metal DFB grating structures^26^ as well as graded photonic heterostructres^27^ provide surface emission and facilitate efficient beam engineering.

Ring QCLs are compact and efficient light sources consisting of a circular resonator comprising a second order DFB grating. The latter provides bi-directional vertical light emission, i.e. surface and substrate emission^28,29^. Recently, a high-power substrate emitting ring QCL was demonstrated^30^. Besides grating-based beam modifications of the surface emission^31^, the bi-directional emission behavior also facilitates shaping of the substrate beam based on manipulation of the bottom side of the chip. An on-chip polarizer fabricated of thin parallel gold wires has been demonstrated to provide a linearly polarized emission beam from a ring QCL^32^. Furthermore, an on-chip gradient index metamaterial can be directly etched into the substrate in order to form a monolithically integrated flat lens^33^. In contrast to plasmonic metasurfaces^34^ or nanopore arrays^35^, this gradient index metamaterial achieves high efficiencies and preserves the coherence of the beam resulting in well-defined interference fringes. However, graphene-based plasmonic metasurfaces with similar efficiencies have been demonstrated^36^.

**Figure 1:**
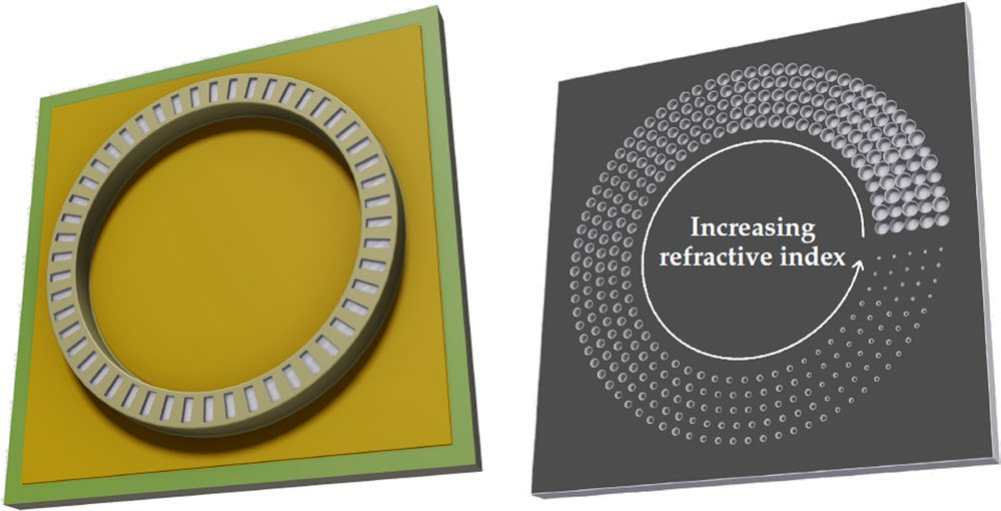
Sketch of the surface (left) and substrate (right) side of the twisted wavefront ring quantum cascade laser (QCL). The gradient index metamaterial consists of differently sized holes directly etched into the substrate. They form a refractive index gradient, which twists the wavefront and generates a helical beam. The shown metamaterial generates a linearly varying phase shift between 0 and 2*π*.

**Figure 2:**
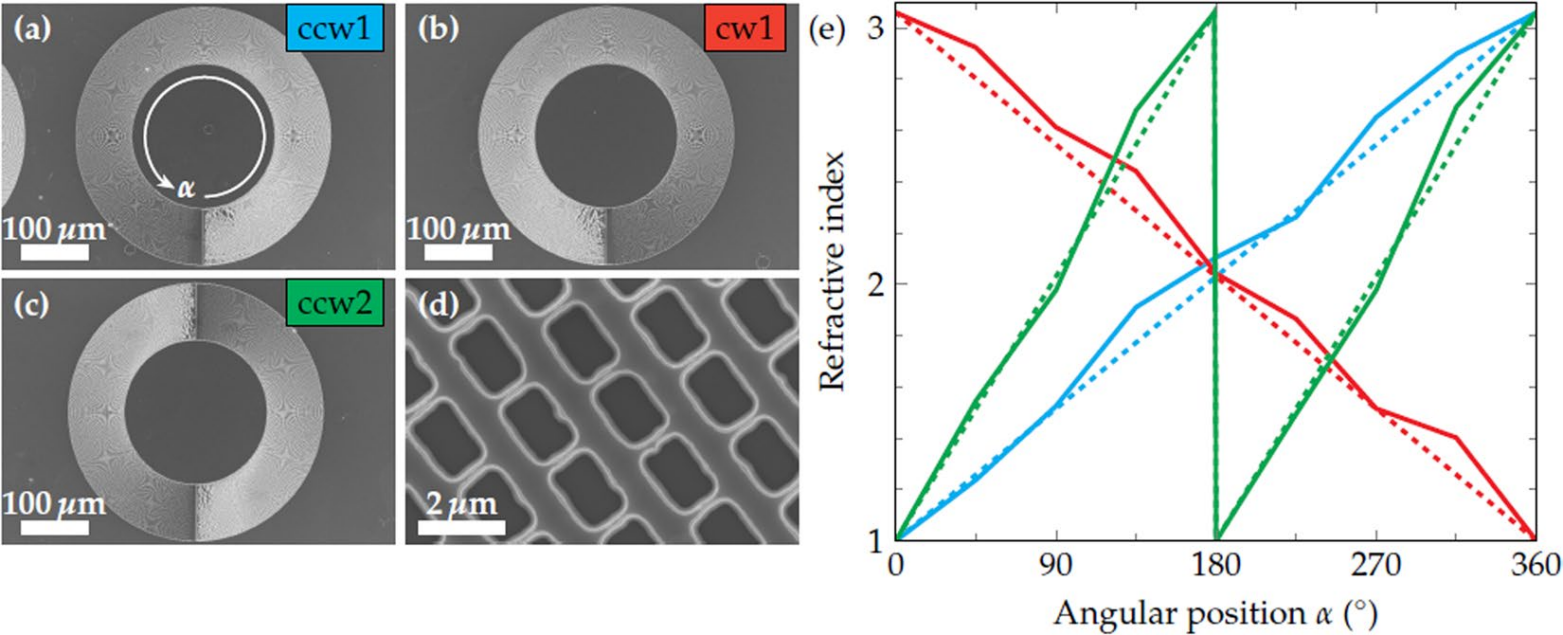
Scanning electron microscopy (SEM) images of gradient index metamaterials with different discontinuity counts. (**a**) Counter-clockwise gradient with a discontinuity count of 1 (ccw1). (**b**) Clockwise gradient with a discontinuity count of 1 (cw1). Counter-clockwise gradient with a discontinuity count of 2 (ccw2). (**d**) Close-up of the etched holes. (**e**) Experimentally determined (solid) and theoretical (dashed) refractive index gradients for different discontinuity counts.

Based on such a gradient index metamaterial, we demonstrate here the on-chip generation of QCL-beams with a twisted wavefront. Such helical wavefronts can be utilized to create OAM beams, which are promising tools in various fields such as sensing of chiral molecules^37^, telecommunications^38^, microscopy^39^ and micromanipulation of microscopic particles^40^. The monolithic generation of OAM beams enables a crucial miniaturization and simplification of setups exploiting the OAM of light^10,41^. Our approach for a QCL with a twisted wavefront is schematically shown in Fig. 1. A conventional ring QCL is fabricated on the surface side of the chip. Thereafter, the gradient index metamaterial, which consists of differently sized holes, is etched into the substrate side according to ref.^33^. In contrast to the on-chip lens, the refractive index exhibits not a radial but an azimuthal gradient. Since this approach requires only a single electron beam lithography exposure and does not depend on gray scale lithography, it is quite reproducible and rather robust. The key aspect is the linear tilt of the wavefront in the azimuthal direction as well as the phase-matching condition, i.e. the step height of the discontinuity of the metamaterial. The latter is given by the etch depth and amounts to *λ*/(*n*_0_ − 1) with the wavelength *λ* = 8.77*μ*m and the refractive index *n*_0_ = 3.06 of the InP substrate. Figure 2(a–c) depict scanning electron microscopy (SEM) images of gradient index metamaterials with different discontinuity counts and gradient directions. In Fig. 2 an increasing refractive index in clockwise and counter-clockwise direction is labeled cw and ccw, respectively. The number in the label refers to the count of discontinuity steps of the metamaterial. In Fig. 2(d) a close-up view of the ccw1 metamaterial is shown. For each of the given metamaterials the refractive index gradient was estimated by measuring the hole sizes as a function of the angular position *α* and considering the aspect ratio dependent etching (ARDE) according to ref.^33^. The refractive index gradient is in good agreement with the theoretical gradient given by the dashed lines. Furthermore, the experimentally realized etch depth amounts to *d*_met_ = 4.32*μ*m $=1.014\cdot \lambda /({n}_{0}-1)$.

**Figure 3:**
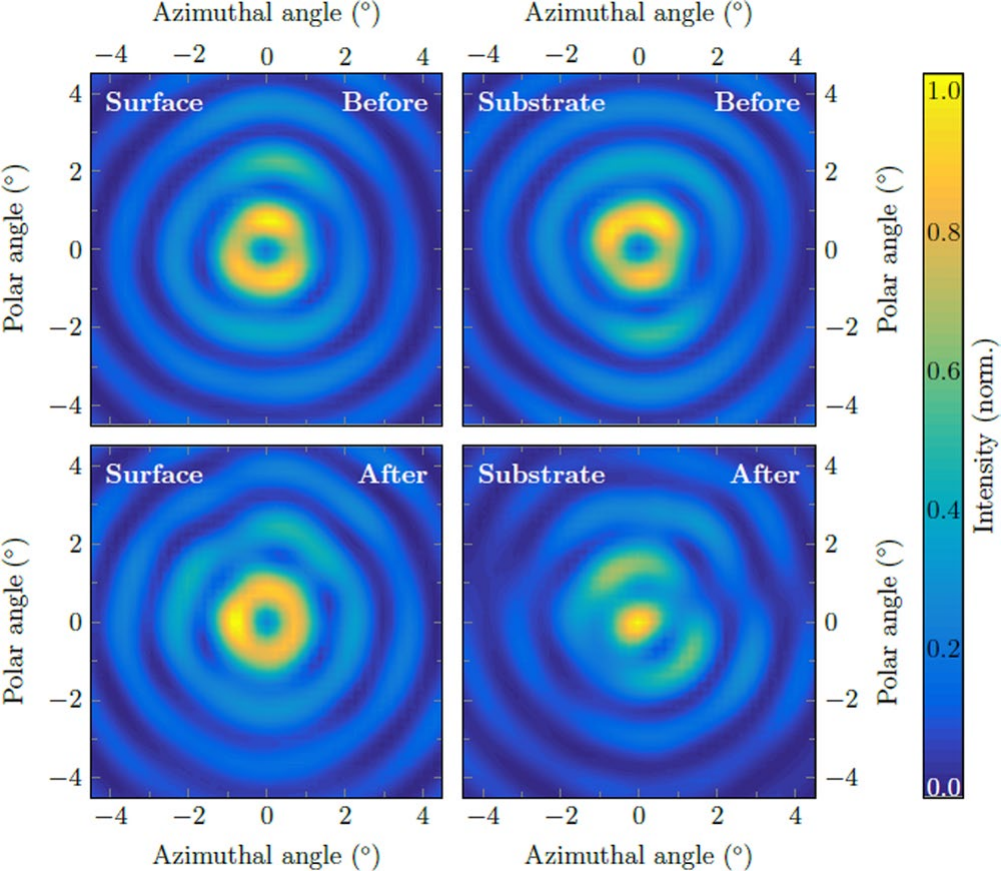
Beam patterns measured with an MCT detector. Surface (left) and substrate (right) emission before (top) and after (bottom) fabrication of the ccw1 metamaterial. For the substrate emission the metamaterial induces a change from a central intensity minimum to a maximum.

**Figure 4:**
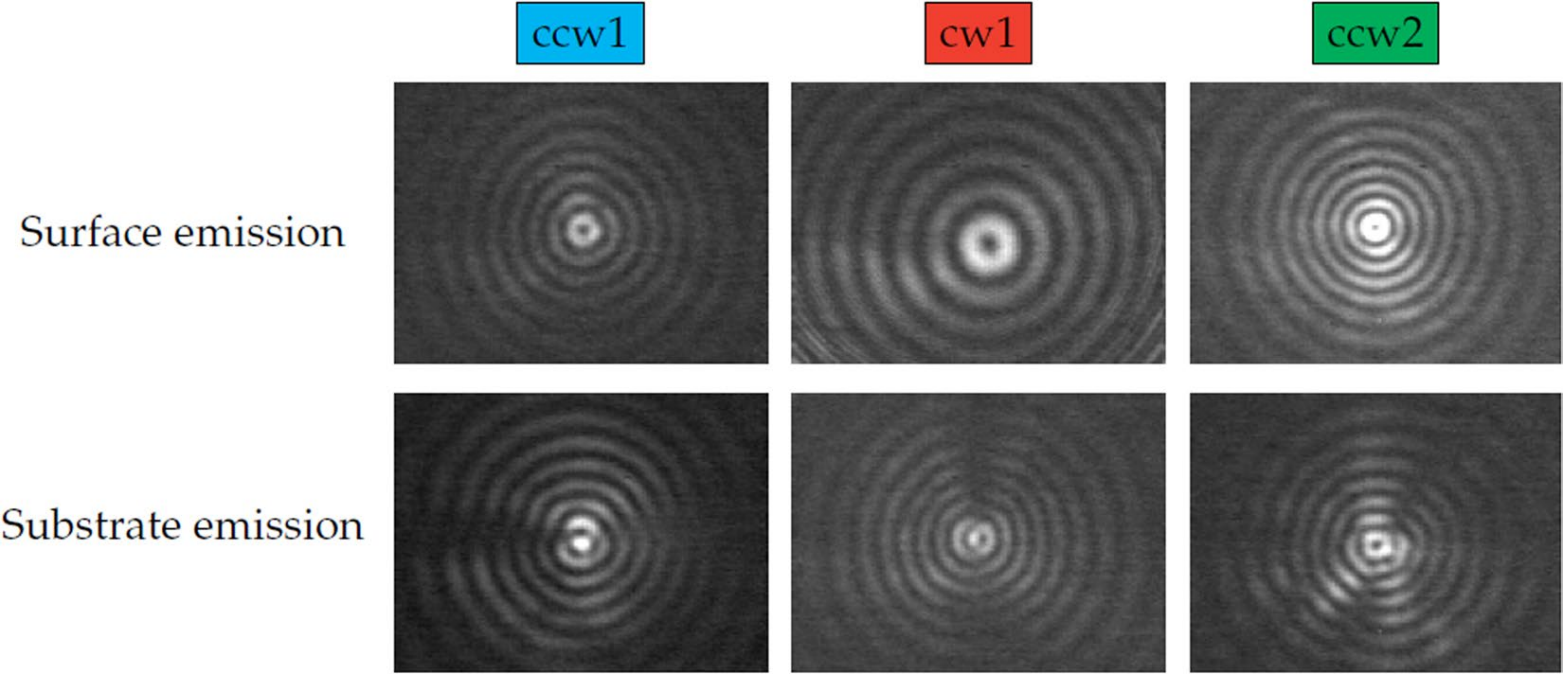
Surface and substrate emission patterns for different metamaterials measured with a bolometer camera. While the ccw1 & cw1 metamaterials generate a central intensity maximum, the beam of the ccw2 metamaterial exhibits a central minimum.

In the following, the effect of the gradient index metamaterial on the emission beam of ring QCLs is investigated. Far fields of the surface and substrate emission before and after the fabrication of the ccw1 metamaterial are shown in Fig. 3. These far fields are measured with a mercury-cadmium-telluride (MCT) detector on a two-dimensional translational stage at a distance of 15 cm without any optical elements between the laser and the detector. Without the metamaterial, both emission directions exhibit a very similar intensity pattern in the far field consisting of concentric interference rings with a central intensity minimum. This minimum arises due to the fact that ring QCLs exhibit azimuthal polarization^42^, which results in an antiparallel orientation of opposing electric field vectors. In the center of the far field this causes destructive interference. After fabrication of the gradient index metamaterial the surface beam is unchanged while the substrate beam shows a clear central intensity maximum. This can be explained by the varying phase-shift along the azimuthal coordinate induced by the metamaterial and the twisted wavefront. For metamaterials with a discontinuity count of 1 (ccw1 & cw1) the phase-shift between opposing sides of the ring is *π*. It results in a parallel orientation of opposing electric field vectors over the entire ring and therefore provides constructive interference and an intensity maximum in the beam center. Figure 4 demonstrates that the creation of a central intensity maximum depends on the discontinuity count of the metamaterial, but not on the direction of the gradient.

Interestingly, the metamaterial with 2 discontinuities (ccw2) generates a far field with a central intensity minimum. Since for this metamaterial the induced phase-shift between opposing sides is 2*π*, the electric field vectors remain in their original orientation, which results in destructive interference in the beam center. Considering this idea of the orientation of the electric field vector at opposing sides of the ring laser suggests that the beam exhibits central intensity maxima and minima for odd and even discontinuity counts of the metamaterial, respectively.

**Figure 5:**
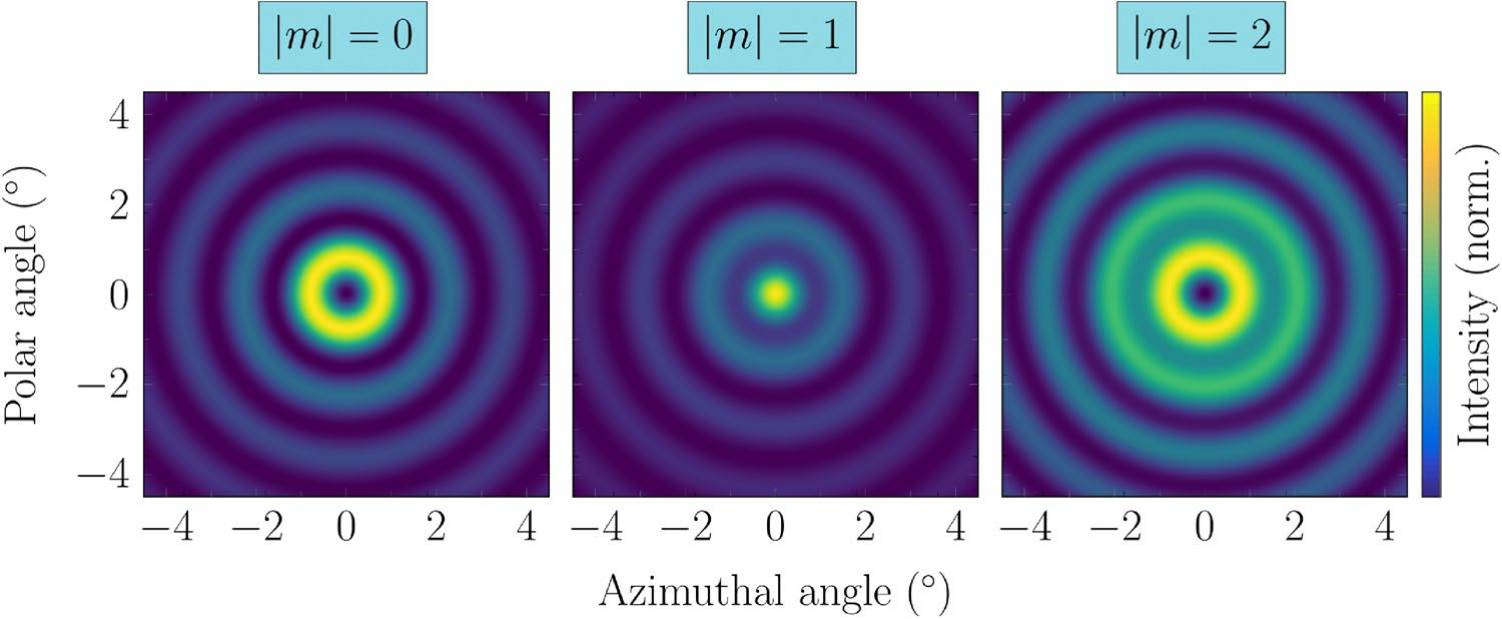
Calculated beam patterns for different topological charges m based on the ray diffraction integral. Since the dipole model produces identical beam patterns they are omitted here.

In order to verify our experimental results we simulated the far field of a ring-shaped emitter with a twisted wavefront. Our theoretical investigation is based on two different approaches. The first method is based on a numerical model featuring 128 azimuthally oriented independent dipoles^22^. The creation of the twisted wavefront is realized by a well-defined phase shift between neighboring dipoles. The second method uses a vectorial ray-based diffraction integral^43^. Here, the twisted wavefront in the simulation is realized by a helical phase plate positioned directly after the ring emitter. The dipole model is based on the Maxwell-Equations while the diffraction integral model is based on the Rayleigh-Sommerfeld diffraction integral. Furthermore, the dipole model consists of 128 discrete dipole emitters while the diffraction integral model assumes a homogeneous emitting area. These theoretical beam patterns feature not only a twisted wavefront but also a well-defined OAM. The two models produce identical far field patterns, which are shown in Fig. 5.

**Figure 6:**
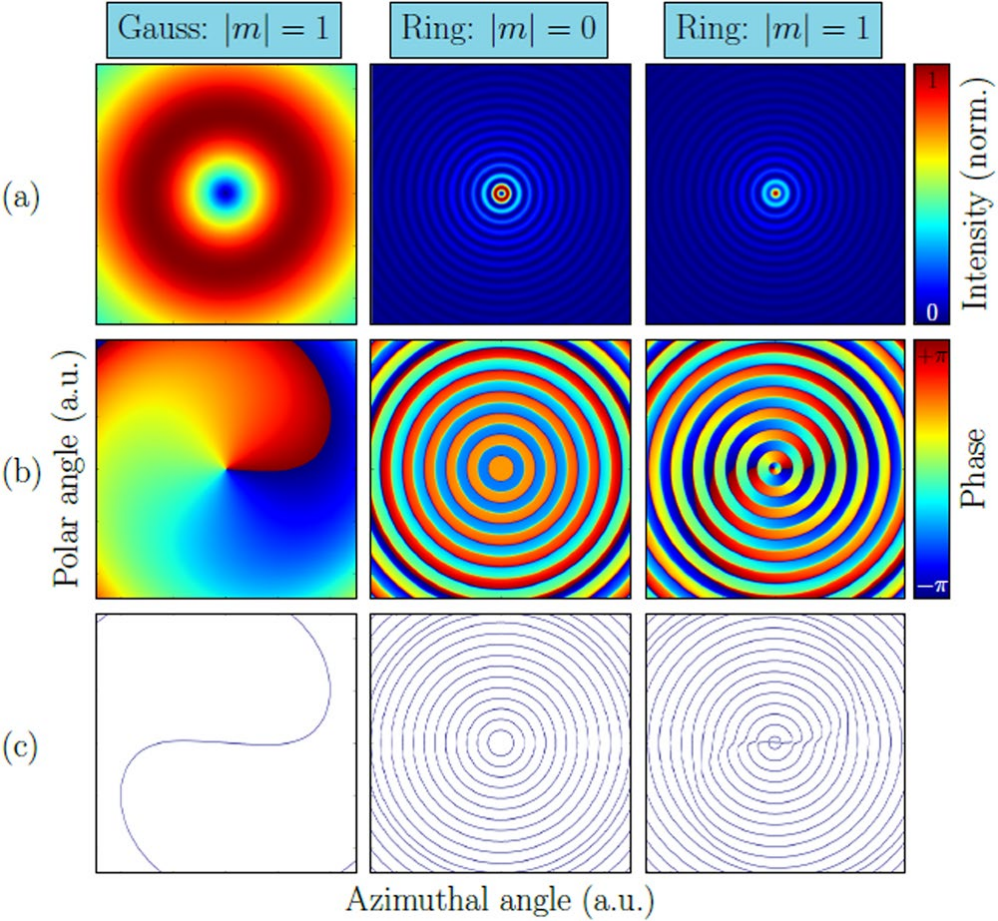
(**a**) Simulated far field patterns (from left to right) of a Gaussian beam, a conventional ring emitter beam and a modified ring emitter beam carrying OAM. (**b**) Phase structure of all the beams in (**a**). Ring emitters exhibit a phase difference of *π* between adjacent interference rings. The OAM phase structure of a ring emitter beam is a mixture of the conventional phase of the ring emitter and the OAM spiral phase pattern of the Gaussian beam. (**c**) Iso-phase lines of the corresponding beams.

This agreement also holds for different discontinuity counts, which, for OAM beams, can be identified with a well-defined topological charge. Ring emitter beams with no OAM (*m* = 0) exhibit a central intensity minimum, while |*m*| = 1 and |*m*| = 2 beams have a central maximum and minimum, respectively. Based on the vectorial ray diffraction integral method, we investigated the phase structure of ring emitter beams with and without OAM and compared it to a Gaussian beam carrying OAM. Our results are shown in Fig. 6. The Gaussian beam exhibits the well-known donut-shaped intensity pattern, while the ring emitter beams show concentric interference fringes with a central minimum and maximum for the conventional and OAM beam, respectively. In Fig. 6(b) the phase of each beam is shown. The OAM Gaussian beam shows a spiral phase pattern, while the non-OAM beam of the ring emitter exhibits a phase difference of *π* between adjacent interference rings. The beam pattern of the OAM ring emitter can be characterized as a mixture between the Gaussian OAM beam and the conventional ring emitter beam. This is also evident from the iso-phase lines shown in Fig. 6(c), which indicate the locations where the phase equals zero.

The agreement between the simulated OAM beams and the experimental twisted wavefront beams suggests that the experimental beams do carry OAM as well. However, this conclusion needs to be validated by a direct experimental proof. In the literature this is often realized in form of an interference experiment^10,16,41^. An OAM and a non-OAM beam coherently interfere with each other and create a spiral interference pattern. We found that this approach is feasible for Gaussian beam profiles but not for beam profiles from a ring-shaped emitter such as a vertically-emitting ring QCL. In our Supplementary Information we show various simulated interference patterns of Gaussian and ring emitter beams. While for the Gaussian beams the well-known spiral interference patterns appear, the corresponding interference patterns of the ring emitter beams do not show spiral patterns. In order to directly prove that our twisted wavefront beams in the experiment in fact carry OAM, a different method, which is suited for ring-shaped emitters, has to be developed. Still, our results clearly show that a direct and controlled manipulation of the emission beam of a ring QCL is possible by means of a spiral gradient index metamaterial. Further research is necessary to identify these beams as OAM beams.

In conclusion, we demonstrate an efficient method for the on-chip generation of twisted wavefront beams from a QCL. Our approach is based on a vertically emitting ring QCL in combination with a gradient index metamaterial, which is directly fabricated into the bottom side of the laser chip. Due to the twisted wavefront the phase relationship along the entire ring is modified, which strongly influences the emission beam. For metamaterials with 1 discontinuity step in the metamaterial the beam pattern changes from a central intensity minimum to a maximum. Metamaterials with 2 discontinuity steps create beams with an intensity minimum in the center. We performed far field simulations of OAM beams, which match our experimental results. However, further research has to be conducted in order to prove *in-situ* that our measured laser beams carry OAM. Our approach could be used for sensing experiments of chiral molecules in the mid-IR^44^.

## Electronic supplementary material

Supplement

## References

[1] El-Ganainy, R. *et al*. Non-hermitian physics and pt symmetry. *Nat. Phys*. **14**, 11 EP, 10.1038/nphys4323 (2018).

[2] Liertzer M (2012). Pump-induced exceptional points in lasers. Phys. Rev. Lett..

[3] Brandstetter, M. *et al*. Reversing the pump dependence of a laser at an exceptional point. *Nat. Commun*. **5**, 4034 EP, 10.1038/ncomms5034 (2014). 10.1038/ncomms5034 PMC4082637 24925314

[4] Peng B (2014). Loss-induced suppression and revival of lasing. Sci..

[5] Feng L, Wong ZJ, Ma R-M, Wang Y, Zhang X (2014). Single-mode laser by parity-time symmetry breaking. Sci..

[6] Hodaei H, Miri M-A, Heinrich M, Christodoulides DN, Khajavikhan M (2014). Parity-time–symmetric microring lasers. Sci..

[7] Wiersig J (2011). Structure of whispering-gallery modes in optical microdisks perturbed by nanoparticles. Phys. Rev. A.

[8] Kim M, Kwon K, Shim J, Jung Y, Yu K (2014). Partially directional microdisk laser with two rayleigh scatterers. Opt. Lett..

[9] Peng B (2016). Chiral modes and directional lasing at exceptional points. Proc. Natl. Acad. Sci..

[10] Miao P (2016). Orbital angular momentum microlaser. Sci..

[11] Mujagić E (2008). Grating-coupled surface emitting quantum cascade ring lasers. Appl. Phys. Lett..

[12] Faist J (1994). Quantum cascade laser. Sci..

[13] Ahn S (2013). Enhanced light output power of quantum cascade lasers from a tilted front facet. Opt. Express.

[14] Ahn S (2014). High-power, low-lateral divergence broad area quantum cascade lasers with a tilted front facet. Appl. Phys. Lett..

[15] Yu N (2008). Quantum cascade lasers with integrated plasmonic antenna-array collimators. Opt. Express.

[16] Yu N, Capasso F (2010). Wavefront engineering for mid-infrared and terahertz quantum cascade lasers. J. Opt. Soc. Am. B.

[17] Hofstetter D, Faist J, Beck M, Oesterle U (1999). Surface-emitting 10.1*μ*m quantum-cascade distributed feedback lasers. Appl. Phys. Lett..

[18] Colombelli R (2003). Quantum cascade surface-emitting photonic crystal laser. Sci..

[19] Sirtori C, Barbieri S, Colombelli R (2013). Wave engineering with thz quantum cascade lasers. Nat. Photonics.

[20] Vitiello MS (2014). Photonic quasi-crystal terahertz lasers. Nat. Commun..

[21] Degl’Innocenti R (2016). Hyperuniform disordered terahertz quantum cascade laser. Sci. Reports.

[22] Schönhuber S (2016). Random lasers for broadband directional emission. Opt..

[23] Sigler C (2014). Design for high-power, single-lobe, grating-surface-emitting quantum cascade lasers enabled by plasmon-enhanced absorption of antisymmetric modes. Appl. Phys. Lett..

[24] Jouy P (2015). Surface emitting multi-wavelength array of single frequency quantum cascade lasers. Appl. Phys. Lett..

[25] Boyle C (2016). High-power, surface-emitting quantum cascade laser operating in a symmetric grating mode. Appl. Phys. Lett..

[26] Liang G (2013). Single-mode surface-emitting concentric-circular-grating terahertz quantum cascade lasers. Appl. Phys. Lett..

[27] Xu G (2012). Efficient power extraction in surface-emitting semiconductor lasers using graded photonic heterostructures. Nat. Commun..

[28] Lyakh A, Zory P, D’Souza M, Botez D, Bour D (2007). Substrate-emitting, distributed feedback quantum cascade lasers. Appl. Phys. Lett..

[29] Schwarzer C (2012). Grating duty-cycle induced enhancement of substrate emission from ring cavity quantum cascade lasers. Appl. Phys. Lett..

[30] Wu DH, Razeghi M (2017). High power, low divergent, substrate emitting quantum cascade ring laser in continuous wave operation. APL Mater..

[31] Szedlak R (2014). Grating-based far field modifications of ring quantum cascade lasers. Opt. Express.

[32] Schwarzer C (2013). Linearly polarized light from substrate emitting ring cavity quantum cascade lasers. Appl. Phys. Lett..

[33] Szedlak R (2014). On-chip focusing in the mid-infrared: Demonstrated with ring quantum cascade lasers. Appl. Phys. Lett..

[34] Yu N (2013). Flat optics: Controlling wavefronts with optical antenna metasurfaces. IEEE J. Sel. Top. Quantum Electron..

[35] Zhang J-C (2014). Directional collimation of substrate emitting quantum cascade laser by nanopores arrays. Appl. Phys. Lett..

[36] Li Z (2015). Graphene plasmonic metasurfaces to steer infrared light. Sci. Reports.

[37] Brullot W, Vanbel MK, Swusten T, Verbiest T (2016). Resolving enantiomers using the optical angular momentum of twisted light. Sci. Adv..

[38] Wang J (2012). Terabit free-space data transmission employing orbital angular momentum multiplexing. Nat. Photonics.

[39] Ritsch-Marte, M. Orbital angular momentum light in microscopy. *Philos. Transactions Royal Soc. Lond. A: Math., Phys. Eng. Sci*. **375**, 10.1098/rsta.2015.0437 (2017). 10.1098/rsta.2015.0437 PMC5247481 28069768

[40] O’Neil AT, MacVicar I, Allen L, Padgett MJ (2002). Intrinsic and extrinsic nature of the orbital angular momentum of a light beam. Physi. Rev. Lett..

[41] Li H (2015). Orbital angular momentum vertical-cavity surface-emitting lasers. Opt..

[42] Bai Y (2011). High power, continuous wave, quantum cascade ring laser. Appl. Phys. Lett..

[43] Andreas B, Mana G, Palmisano C (2015). Vectorial ray-based diffraction integral. J. Opt. Soc.Am. A.

[44] Lambrecht A, Pfeifer M, Konz W, Herbst J, Axtmann F (2014). Broadband spectroscopy with external cavity quantum cascade lasers beyond conventional absorption measurements. Analyst.

