# Supplementary material for: Ring quantum cascade lasers with twisted wavefronts
Source: Sci Rep. 2018 May 22;8:7998. doi: 10.1038/s41598-018-26267-x (PMC5964118; doi:10.1038/s41598-018-26267-x)
Supplement: Supplementary file 1 — Supplement [file 41598_2018_26267_MOESM1_ESM.pdf]

Supplementary Information: Ring quantum cascade lasers with twisted wavefronts

Rolf Szedlak[1,*], Thomas Hisch[2], Benedikt Schwarz[1], Martin Holzbauer[1], Donald MacFarland[1], Tobias Zederbauer[1], Hermann Detz[3], Aaron Maxwell Andrews[1], Werner Schrenk[1], Stefan Rotter[2], and Gottfried Strasser[1]

[1]Institute of Solid State Electronics & Center for Micro- and Nanostructures, TU Wien, Floragasse 7, 1040 Vienna, Austria
[2]Institute for Theoretical Physics, TU Wien, Wiedner-Hauptstraße 8-10/136, 1040 Vienna, Austria
[3]Austrian Academy of Sciences, Dr. Ignaz Seipel-Platz 2, 1010 Vienna, Austria
[*]rolf.szedlak@tuwien.ac.at

ABSTRACT

We demonstrate the on-chip generation of twisted wavefront beams from ring quantum cascade lasers. A monolithic gradient index metamaterial is fabricated directly into the substrate side of the semiconductor chip and induces a twist of the light's wavefront. This significantly influences the obtained beam pattern, which changes from a central intensity minimum to a maximum depending on the discontinuity count of the metamaterial. Our design principle provides an interesting alternative to recent implementations of microlasers operating at an exceptional point.

Figure 1 shows the simulated interference patterns of Gaussian orbital angular momentum (OAM) beams with different topological charges.

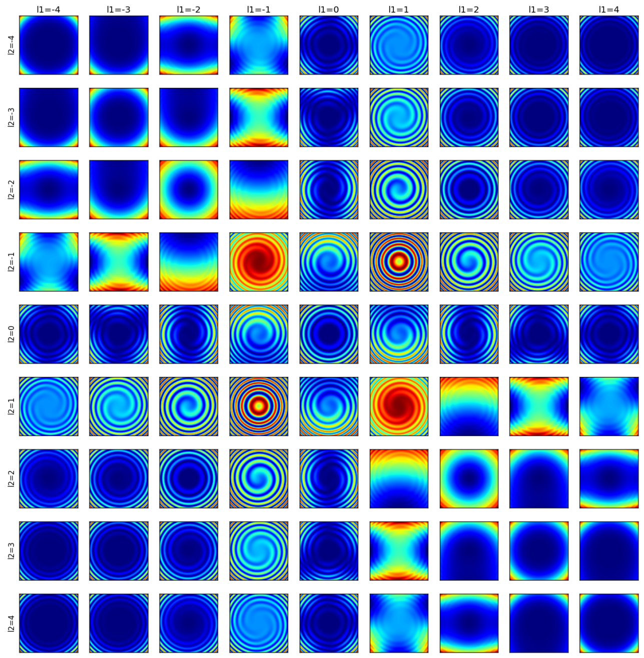

Figure 1. Interference pattern of Gaussian beams with different topological charges. Distinct spiral patterns are visible.

Figure 2 shows the simulated interference patterns of ring emitter orbital angular momentum (OAM) beams with different topological charges.

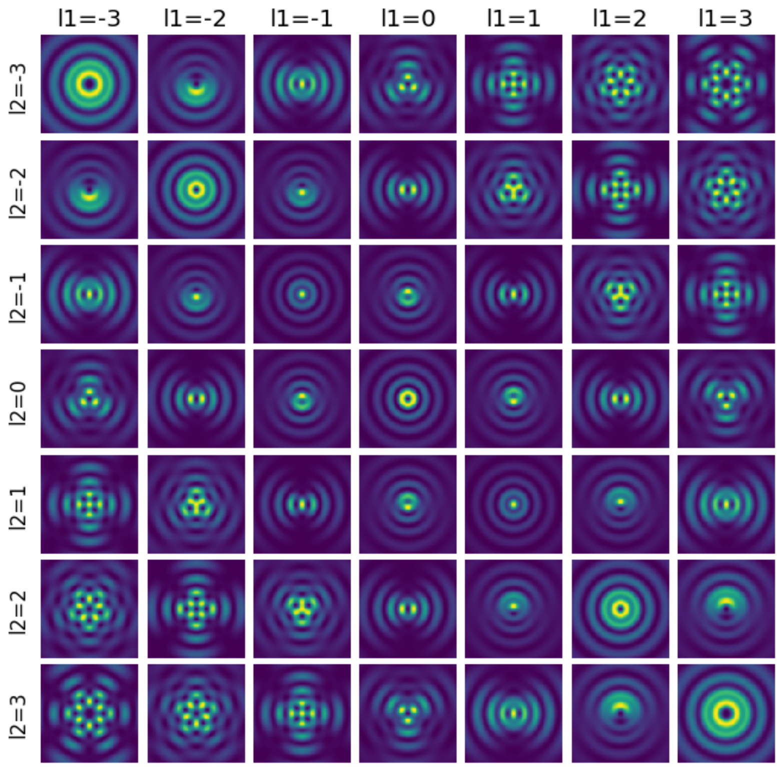

Figure 2. Interference pattern of ring emitter beams with different topological charges. No spiral patterns are visible.
